# Application of mesenchymal stem cell sheet to treatment of ischemic heart disease

**DOI:** 10.1186/s13287-021-02451-1

**Published:** 2021-07-07

**Authors:** Dehua Chang, Taibing Fan, Shuang Gao, Yongqiang Jin, Mingkui Zhang, Minoru Ono

**Affiliations:** 1 Department of Cell Therapy in Regenerative Medicine, The University of Tokyo Hospital, 7-3-1 Honggo, Bunkyo-ku, Tokyo, 113-8655 Japan; 2 Children Heart Center, Fuwai Central China Cardiovascular Hospital, No.1 Fuwai Road, Zhengzhou, 450018 China; 3 Research and Development Department, BOE Regenerative Medicine Technology Co., Ltd., NO.9 JiuXianQiao North Road, Beijing, 100015 China; 4 Heart Center, First Hospital of Tsinghua University, NO.6 JiuXianQiao 1st Road, Beijing, 10016 China; 5 Department of Cardiac Surgery, The University of Tokyo Hospital, 7-3-1 Honggo, Bunkyo-ku, Tokyo, 113-8655 Japan

**Keywords:** Cell sheet, Cardiac tissue, Cell therapy, Mesenchymal stem cells

## Abstract

In recent years, mesenchymal stem cells (MSCs) have been used to improve cardiac function and attenuate adverse ventricular remodeling of the ischemic myocardium through paracrine effects and immunoregulation functions. In combination with cell sheet technology, MSCs could be more easily transplanted to the ischemic area. The long-term retention of MSCs in the affected area was realized and significantly improved the curative effect. In this review, we summarized the research and the applications of MSC sheets to the treatment of ischemic heart tissue. At present, many types of MSCs have been considered as multipotent cells in the treatment of heart failure, such as bone marrow-derived mesenchymal stem cells (BM-MSCs), adipose-derived mesenchymal stem cells (AD-MSCs), umbilical cord-derived mesenchymal stem cells (UC-MSCs), and skeletal myoblasts (SMs). Since UC-MSCs have few human leukocyte antigen-II and major histocompatibility complex class I molecules, and are easy to isolate and culture, UC-MSC sheets have been proposed as a candidate for clinical applications to ischemic heart disease.

## Introduction

Heart failure is the leading cause of death worldwide, despite improvements in management through mechanical and surgical therapeutic strategies. Heart transplant is the standard curative therapy for end-stage heart failure. However, the shortage of donor hearts limits the application of this procedure, which also requires lifelong immunosuppression. Other surgical interventions, such as the implanting of mechanical ventricular assist devices, have high associated medical costs and can easily cause bleeding, infection, and other complications. Recently, stem cell therapy has been developed and extensively investigated as an alternative therapeutic strategy for heart failure. It is well-known that heart tissue is difficult to repair itself, and adult cardiac cells tend not to divide, so it may be possible to repair ischemic areas by transplanting mesenchymal stem cells (MSCs). MSCs have been used to treat damaged hearts as they offer a low immunogenic property, paracrine effect, and immunosuppression. MSCs secrete growth factors and chemokines which induce cardio-protection, stimulate angiogenesis, and reorganize the extracellular matrix in ischemic scar areas. Several clinical studies have described how the use of MSCs improved cardiac function and the regeneration of heart tissue in damaged hearts [1–3].

High colonization rates and long survival times after transplanting are two key points in the curative effects of MSCs. Suspensions of MSCs injected into the ischemic area of the heart often present underwhelming results, because of cell loss and a low retention of transplanted cells. Cell sheet technology eliminates the problem of retention, as all MSCs remain at the implantation site. In addition, a cell sheet is a scaffold-free composition of cells, avoiding the potential inflammatory response to scaffold degradation.

A cell sheet engineering technology was developed by Okano’s team, using thermo-responsive culture dishes coated with poly (N-isopropylacrylamide) (PIPAAm) [4, 5]. With this application, changes in culture temperature lead to oscillation between hydrophilic and hydrophobic states. Cells adhere to and proliferate on a culture dish surface at 37°C, and a monolayer cell sheet with an extracellular matrix (ECM) detaches spontaneously at temperatures lower than 32°C, without enzymatic digestion [6, 7]. The cell sheet is scaffold-free and provides a new method of cell implantation. The technique is indispensable for harvesting cell sheets, selecting cell sheet types, and applying cell sheets.

Normally in cell therapy, suspension cells are used though injection and intravenous infusion. Studies have hypothesized that poor retention as a result of blood flow causes decreased effectiveness, because it is a zero-dimensional application [8]. A cell sheet based on a two-dimensional cell culture is a more promising approach because of its scalability and accessibility. The cell surface proteins and the ECM are preserved in the cell sheet and make it possible to paste easily in situ. This improves the using ratio and effect, compared to dissociated cell injection as reported in previous studies [9, 10]. In addition, cell sheets can grow into a three-dimensional (3D) cell-dense tissue construct by the sequential stacking of confluent cell sheets [11]. Sekine et al. fabricated a cardiac cell sheet from newborn rats and laid four sheets on top of each other until they fused. The layered cardiac cell sheet was then implanted under the skin of immunodeficient rats. The engineered cardiac tissue was beating and the blood vessels had permeated it after 6 months [12–14]. Most bioreactors cannot supply enough nutrients and oxygen to the growing tissue. Sakaguchi et al. reported on the preparation of 3D tissues using cell sheets and a perfusion bioreactor having collagen-based microchannels. Cardiac cell sheets were incubated, and endothelial cells migrated to vascularize in the collagen and connect with the microchannels. When an additional triple-layer sheet was prepared, engineered vascularized cardiac tissue was successfully fabricated in vitro. This suggests it is possible for cell sheets to be used in a block shape in the regeneration of tissue or organ defects [11].

In this review, we mainly summarized the application of MSC-derived cell sheets on the ischemic heart tissue. MSCs from various tissues share many characteristics. Several types of MSC sheets were classified, and the characteristics and applications of each kind of cell were analyzed. We also summarized the research and applications of human-induced pluripotent stem cell-derived cardiomyocytes (iPS-CM) in pre-clinical study.

## MSC-derived cell sheets

MSCs are adult stem cells that can be isolated from various tissues. They originate from mesodermal lineages and have been differentiated into connective tissues, such as osteoblasts, chondrocytes, cardiomyocytes, and adipocytes [15]. The International Society for Cellular Therapy has proposed a set of standards to define MSCs [16, 17]. As above, MSCs present in many kinds of tissue. They were isolated and cultured in vitro, which makes possible their large-scale proliferation. With the addition of widely recognized security, MSCs are presently being used in cardiovascular work and in other fields [2, 18–20]. Many studies have shown that MSCs can improve cardiac function through their immunomodulatory properties, paracrine secretion, and cell–cell interactions. They can reduce inflammation, protect surrounding tissue, and stimulate vascularization to nourish cardiomyocytes after ischemic injury of the heart [21]. The studies indicate that control of infarct size and the cessation of ventricular remodeling is a compensated process for the attenuated ventricular ejection property [22]. Thus, unlike pharmacologic and surgical approaches, cell therapy can stimulate endogenous tissue regeneration to reverse worsening cardiac dysfunction. Most cell therapy for ischemic heart is cell suspension injection (including trans-endocardial and intramyocardial injections, and intracoronary infusion) and intravenous infusion [23]. It may be possible to repair small ischemic areas by injecting MSCs into the tissue, but repairing large damaged areas requires laboratory-grown cell sheet patches [24]. Kim et al. have shown that cell sheet transplantation in situ promoted cellular engraftment and upregulated the growth factor and cytokine expression, compared to intramyocardial injection in a rat myocardial infarction (MI) model [9]. Other research showed that the donor cell survival rate in cell sheet transplantation was more than 11 times higher than for MSC injection, after 28 days [25]. Table 1 summarizes the animal studies on the administration of MSC sheets.

**Table 1:** Administration of MSC sheets in animal models

Cell type	Cell source	Disease model	Animal species	Number of cells	Groups and main result*	Reference
BM-MSC	Rat	MI	Rat	1.2 × 10^7^, two sheets	VEGF BM-MSC sheet (++); BM-MSC sheet (+); Negative control	Augustin et al. [26]
BM-MSC	Rat	MI	Rat	4.0 × 10^6^, one sheet	BM-MSC sheet (+) Negative control	Narita et al. [10]
BM-MSC	Human	ICM	Porcine	1.0 × 10^8^, 10 sheets	BM-MSC sheet (+) Negative control	Kawamura et al. [27]
BM-MSC	Rabbit	MI	Rabbit	-	Hypoxic conditioned BM-MSC sheet (++) BM-MSC sheet (+) Negative control	Tanaka et al. [28]
BM-MSC	Rat	MI	Rat	4.0 × 10^6^, one sheet	Autologous BM-MSC sheet (+) Allogeneic BM-MSC sheet (+) Negative control	Tano et al. [29]
AD-MSC	Rat	MI	Rat	~9.0 × 10^6^, one sheet	AD-MSC sheet (+) Dermal fibroblasts sheet Negative control	Miyahara et al. [30]
AD-MSC	Human	MI	Rat	-	AD-MSC sheet (+) Cardiomyoblast-like sheet (++) Negative control	Okura et al. [31]
AD-MSC	Porcine	HF	Porcine	Three-layered sheets	AD-MSC sheet (+) Negative control	Ishida et al. [32]
AD-MSC	Mouse	MI	Mouse	One sheet	AD-MSC sheet (+) Intraperitoneal injection (+) Negative control	Kim et al. [9]
UC-MSC	Human	MI	Mouse	~1.0 × 10^6^, one sheet	UC-MSC sheet (++) UC-MSC suspension (+) Negative control	Guo et al [33]

*The symbols (+) and (++) indicate the result of the individual study only

*MI* myocardial infarction, *ICM* ischemic cardiomyopathy, *HF* heart failure

### Bone marrow-derived mesenchymal stem cell (BM-MSC) sheets

BM-MSCs are one of the most popular cell sheets for use in cases of MI, because of their convenient isolation and culture in vitro. BM-MSC populations were increased through the repeated passages of the cells by trypsinization. In primary culture, non-adherent cells were removed by medium changes, and the remaining purified BM-MSCs were further expanded in continuous culture. Usually, purity will improve after two or three passages [15].

Narita et al. reported that syngeneic BM-MSC sheets transplanted onto the heart epicardium had effective retention within 1 month, compared to intramyocardial injection in an MI rat model [10]. The initial boost of exogenous BM-MSCs amplified subsequent paracrine effects to regenerate damaged heart tissue [10]. With the application of allogeneic BM-MSC sheets, immunologic response should be balanced against therapeutic effects. Tano et al. showed that allogenic BM-MSC sheets transplanted onto rat heart tissue did evoke low-level immunologic response, characterized with accumulation of CD4^+^ cells and upregulation of IL6. However, substantial numbers of allogenic BM-MSCs survived for a certain early period and improved cardiac function [29].

A cell sheet improves the efficiency of transplanted BM-MSCs. Research into BM-MSC sheet adhesion over time to porcine heart tissue showed that the basal side of the cell sheet has more ECM components, including some collagens, fibronectin, and specialized proteins, making it more likely to adhere to the surface of heart tissue without any mechanical device such as suturing. Chang et al. identified a minimum time of 30 min for adhesion of the basal side of a cell sheet to heart tissue [34]. To enhance the angiogenic property of BM-MSCs, some researchers remolded BM-MSCs or BM-MSC sheets [26, 28]. A cell sheet fabricated by vascular endothelial growth factor (VEGF)-A gene transduced BM-MSCs showed a better result in improving capillary density in the infarct border zone and reducing infarct size in a rat MI model [26]. Tanaka et al. undertook 48 h of hypoxic preconditioning to improve VEGF secretion of autologous BM-MSC sheets, which showed higher therapeutic efficacy than standard cultured sheets in a rabbit MI model [28]. BM-MSC sheets were also transplanted to ischemic cardiomyopathy (ICM), and showed good curative results in an ICM rat model, with an improvement in left ventricular ejection fraction (LVEF) of 6% at 28 days after the transplant [25]. The effectiveness of BM-MSC sheets was studied in a porcine ICM model with an immunosuppressed drug [27]. After 8 weeks, it was found that the remodeling of the left ventricular (LV) was attenuated, and LVEF was improved by nearly 20% [27].

### Adipose-derived mesenchymal stem cell (AD-MSC) sheets

The adipose tissue has also been considered as a source of MSCs for use in cell therapy, because it is one of the most abundant human tissues. Zuk et al. identified the existence of MSCs with self-renewal and multipotent capacities in adipose tissue [35]. The isolating of AD-MSCs relies on collagenase digestion followed by centrifugal separation to isolate the stromal vascular fraction from primary adipocytes [36].

Similar to BM-MSCs, these studies showed that the use of AD-MSC sheets preserves cardiac function, improves cardiac perfusion, and reduces scar tissue size and cardiac remodeling, compared to intramyocardial injection [37]. Kim et al. showed that AD-MSC sheets led to similar improvement in LV systolic function with infarct zone injection, improved cellular engraftment, growth factor, and cytokine expression, which ultimately attenuated adverse cardiac remodeling [9]. As for effectiveness, Miyakawa et al. reported on the application of AD-MSC sheets for improved heart function [30]. The objective of this study was to evaluate the in vivo regenerative potential of AD-MSC sheets in a rat model. The researchers also described an in vivo study of transplantation. The results showed that the AD-MSC sheets gradually grew to a thick stratum with vessels, triggered angiogenesis through paracrine, and improved cardiac function through reversing left ventricle wall thinning.

AD-MSCs are also used to co-culture with other cells and to prepare multilayered cell sheets. Sasagawa et al. used double-layered AD-MSC sheets sandwiching pre-vascularized human umbilical vein endothelial cells (HUVECs) to form multilayered cell sheets [38]. The existence of HUVEC could make a rapid connection to the host vasculature in the early post-transplant period [38]. Besides prefabricating vessels in layered cell sheets, differentiation of human AD-MSCs into cardiomyoblast-like cells was conducted by dimethyl sulfoxide inducing for 48 h [31]. Transplantation of AD-MSCs induced cardiomyoblast-like cell sheets also resulted in recovery of cardiac function and an improved cell survival rate [31]. Ishida et al. studied triple-layered AD-MSC sheets in a porcine chronic heart failure model. The research results showed that transplantation of AD-MSC sheets improved cardiac function and induced newly formed vessels [32].

### Umbilical cord-derived mesenchymal stem cell (UC-MSC) sheets

UC-MSCs are appearing in cardiac tissue engineering, with the assistance of differentiating agents. UC-MSCs are isolated from Wharton’s jelly of the umbilical cord, by enzyme digestion or explant method [39]. UC-MSCs are “zero years old” cells. Their proliferative capacity and cell viability are best compared to BM-MSCs and AD-MSCs, whose performance is significantly affected by the donor’s age. They can be frozen and stored long-term. Guo et al. evaluated the feasibility, safety, and therapeutic efficacy of human UC-MSC sheets in mouse MI models. Results showed that UC-MSC sheets significantly improved retention and survival, compared with UC-MSCs in suspension. UC-MSC sheets also significantly promoted angiogenesis, reduced fibrosis, and modulated post-MI inflammation, thereby improving cardiac function. Researchers also found that inflammation was modulated by decreasing Mcp1-positive monocytes and CD68-positive macrophages and increasing Cx3cr1-positive non-classical macrophages [33]. Recently, the BOE Regenerative Medicine Technology team has verified the effectiveness of human UC-MSC sheets in a porcine chronic myocardial ischemia model. Results showed that human UC-MSC sheets can promote cardiac function and reduce the fibrosis area of the LV. From data collected, the LVEF of the UC-MSC sheet implantation group improved from 42.25 ± 1.23% to 66.91 ± 1.10% at 9 weeks after implantation, while the LVEF of the sham operation group decreased from 42.52 ± 0.65% to 39.55 ± 1.97 at the ninth week. For safety evaluation, oncogenicity, tumor enhancement, diffusion after implantation, and acute and short-term toxicity studies were conducted. They demonstrated that UC-MSC sheets are safe as a cell drug for ischemic myocardium. These researches are not only an important scientific result, but also a big step in the investigation of the UC-MSC sheet as a new drug.

Compared to BM-MSC and AD-MSC sheets, the number of reports on the UC-MSC sheet is limited. However, its advantages and clinical application potential have attracted more and more attention from researchers [40–42]. Nakao et al. used BM-MSCs, AD-MSCs, and UC-MSCs to fabricate cell sheets and compared their proliferation properties and cytokine secretion conditions [41]. The results showed that UC-MSC sheets had a higher proliferation rate and secreted more PEG_2_ and IL-6 than the other two kinds of cell sheets [41]. UC-MSC sheets can be spontaneously pasted onto immune-deficient mouse subcutaneous tissue within 10 min of placement [42] and can remain on ectopic target and promote blood vessel formation. This indicates that UC-MSCs may have continuous cytokine secretion in vivo [42].

Human UC-MSCs are a non-intrusive acquisition with good ethical availability as a population of multipotent cells [43]. They are abundant in the human tissue and provide several possible benefits to ischemic heart tissue. Additionally, the establishment of a human UC-MSC cell bank with a low risk of viral contamination, providing safe allogeneic human UC-MSCs, is expected to provide an off-the-shelf product that can further expand their clinical application [44].

## Other types of cell-derived cell sheet

Although MSCs have been used to produce cell sheets for the heart tissue repair because of their cardiomyocyte differentiation potency and paracrine action, it will be some time before MSC sheets are used clinically. Skeletal myoblast (SM) sheets and induced pluripotent stem cell (iPSC) sheets have already been used in patients, pioneering the clinical application of cell sheets in ischemic heart disease.

### SM-derived cell sheet

SMs are derived from a satellite cell or cell lying beyond the myofiber, with a similar method of differentiation of cardiomyocyte [45, 46]. There are numerous studies of SM transplantation to the myocardium to improve cardiac function [47], and the use of SM sheets to repair damaged cardiac muscle in cardiomyopathy [48]. After implanting SM sheets, angiogenesis, the recovery of diastolic function, and increased wall thickness were observed. These may be the major components of the regenerative mechanism [49]. The modification of myoblast over expression of the *bcl2* gene or hepatocyte growth factor (HGF) gene were studied in a rat model and showed a higher improvement in heart function [50–52]. Autologous SM sheets were directly transplanted onto a patient’s heart tissue. In 2006, Sawa et al. reported a case in which a 56-year-old man with severe dilated cardiomyopathy (DCM) received an autologous SM sheet transplant in a clinical study [53]. Three months later, LVEF had improved from 26% to 46%, and the patient was finally weaned from a LV assist system [53]. Since the cell sheets were produced by the patient’s own skeletal muscle cells, transplantation caused no rejection. After that, Sawa’s group performed clinical research on four patients with artificial hearts due to DCM, and seven patients with severe heart failure such as DCM and ICM [54]. No severe ventricular arrhythmia or other adverse events occurred in the 26 weeks after transplantation [54]. LVEFs improved to 7.1 ± 2.8%, the 6-min walking distance increased from 410.1 ± 136.1 m to 455.4 ± 103.7 m, and six patients improved by at least one class under the New York Heart Association functional classes [54]. The results of a larger clinical trial showed that, compared to nonischemic etiology, SM sheets had better clinical effects in patients with ischemic etiology [55]. In 2020, Sawa et al. published a case report on a 3-year-old child with DCM, demonstrating the feasibility and safety of autologous SM sheets [56]. A 6-month follow-up showed that SM sheets can potentially help to maintain and improve the patient’s cardiac function and clinical status [56].

### Pluripotent stem cell-derived cardiac tissue sheet

The differentiation ability of MSCs and SMs has been considered too limited to produce sufficient functioning cardiomyocytes for heart tissue repair. Pluripotent stem cells, including embryonic stem cells (ESCs) and iPSCs, are highly advantageous cell sources for cell sheets, for regeneration of cardiac myocytes (CMs).

Matsumoto et al. fabricated a cell sheet composed of CMs, endothelial cells, and mural cells differentiated for ESCs. They demonstrated that a multilayered sheet attenuated cardiac dysfunction at 1 week after transplantation in a rat model, due to neovascularization mediated by paracrine effects. However, the engraftment efficiency of the ESC-derived cardiac tissue sheet was rather low at 4 weeks after transplantation [57]. Compared to ESCs, iPSCs can be generated from autologous somatic cells without ethical problems for personalized therapy. iPSCs can differentiate into various types of cells, such as cardiomyocytes or other cardiovascular cell types [58]. Their differentiation can be regulated by culture conditions in vitro by cytokine-based or small molecule-based methods [59, 60].

iPSC sheets were transplanted onto myocardial infarcts in an ICM porcine model and a rat MI model [61–63] and showed improvement in cardiac function [61]. An iPSC sheet can improve LVEF, attenuate LV remodeling, and increase neovascularization. This culture system could be the basis for clinical application of human iPSCs, but the risk of teratoma formation remains a major concern, and little evidence of electrical integration between the grafted and host heart tissues could be identified, as the origins of the cells vary considerably [61].

When using iPSC sheets in combination with the omentum flap, the cell survival percentage on an implanted iPSC sheet can be improved significantly because of increased angiogenesis [64, 65]. Besides that, Masumoto et al. fabricated a cell sheet consisting of iPSC-derived cardiomyocytes, endothelial cells, and vascular mural cells [57, 66]. The addition of the other two types of iPSC-differentiated cells enhanced the neovascular formation ability, which enhanced the cell sheet’s structural integrity [57]. Soluble factors secreted from MSCs were also found to be able to promote the maturation of iPSC-derived cardiomyocytes [67]. The cell sheets consisting of a mixture of iPSCs and MSCs showed longer survival and enhanced therapeutic effects in vivo.

Beyond enhancing angiogenesis, other basic researches were conducted [67]. Elimination of the remaining undifferentiated iPSC cells could be achieved under a methionine-free culture condition [68]. Thicker, beating human myocardial tissue with connectable vessels could be fabricated by multilayered iPSC sheets after multi-step transplantation, which suggested the possibility of producing human cardiac tissue [69]. Sawa et al. reported transplants of iPSC-based heart cells in the world’s first clinical trial in January 2020. The iPSCs were derived from a healthy donor’s blood cells and stored. iPSC sheets attached to the heart’s surface are expected to grow and to secrete a protein that can regenerate blood vessels and improve cardiac function. We look forward to the report on further therapeutic effects after iPSC sheet transplantation.

iPSC-derived MSCs have become another cell source for MSC therapies in recent years. Compared to BM-MSCs, iPSC-derived MSCs have better proliferative capacity and differentiation potential, being little affected by cellular senescence. Research has demonstrated that the immunomodulation and paracrine effects of iPSC-derived MSCs can improve the cardiac function of a porcine HF model [70]. iPSC-derived MSCs also showed promising potential in cardiovascular disease caused by anthracyclines, cigarette smoke, and atherosclerosis [71–73]. There has been little research into combining iPSC-derived MSCs and cell sheet technology. We believe that related future research would be fruitful.

## Discussion

**Fig. 1:**
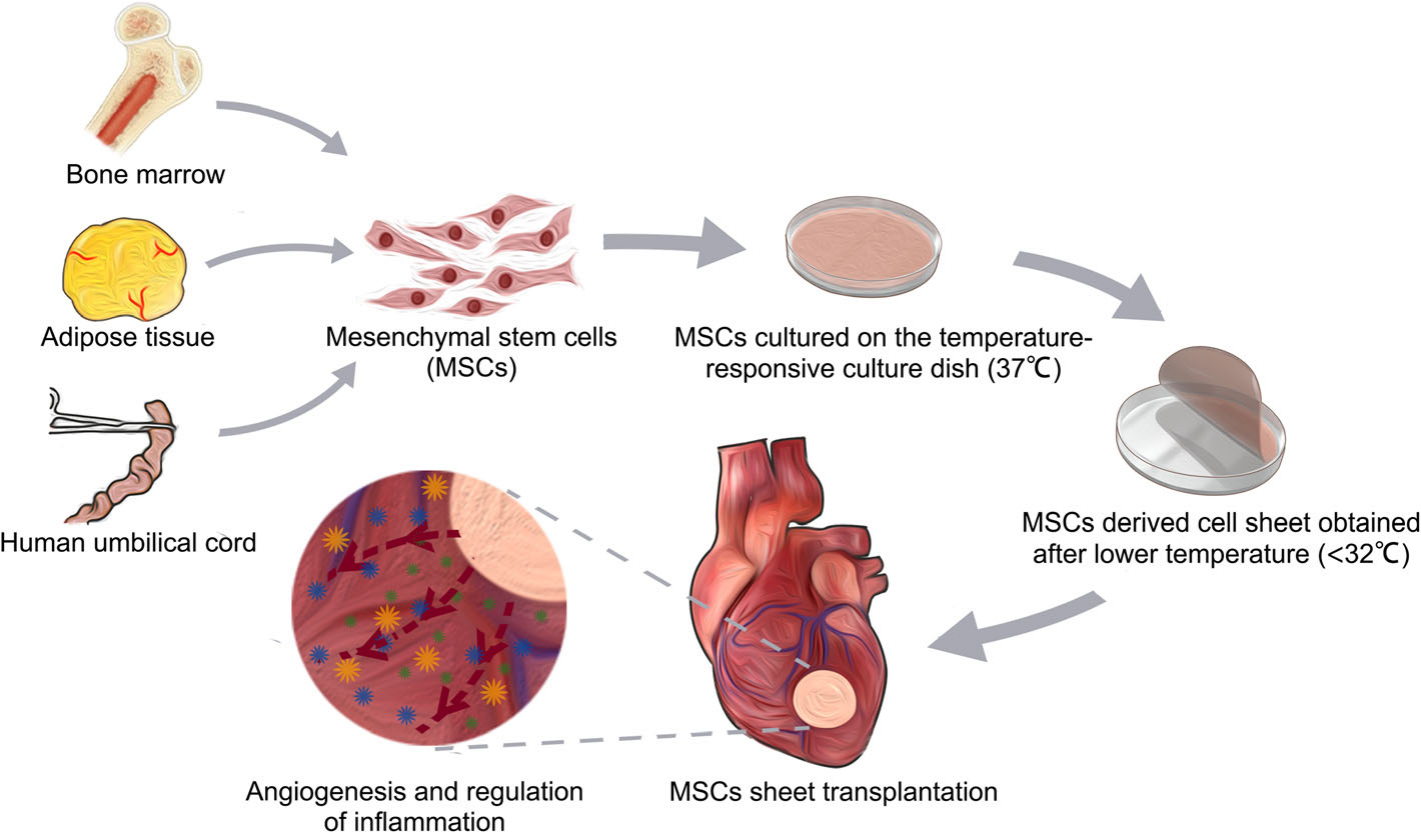
Cell sheet fabrication by MSCs from different tissue sources, and the effects of MSC sheets on ischemic heart disease.

In this review, we have described the application of many types of cell sheets in the repair of ischemic heart tissue. Cell sheets from various cell sources can be fabricated and transplanted to improve cardiac function. However, the high cost and the time required to manufacture each patient-derived cell sheet hinder the dissemination of cell sheet-based regenerative medicine. Figure 1 is a schematic of cell sheets fabricated by MSCs from the bone marrow, adipose tissue, and umbilical cord. The main mechanisms of action underlie the favorable actions of MSCs in ischemic heart disease: (1) stimulation of angiogenesis [41], (2) reduction of fibrosis, (3) anti-inflammatory and immunomodulatory activities [74], and (4) the release of signaling molecules of injury and capture of the stem cells, which are involved in proliferation, migration, differentiation, and engraftment in the ischemic tissue [75]. After a cell sheet is transplanted onto the infarct surface of the left ventricle, angiogenesis and inflammation regulation are achieved by paracrine, which is the universally recognized mechanism. Angiogenesis promoting of MSCs was achieved by secreting CXCL1, CXCL5, CXCL6, CXCL8, HGF, and other cytokines [76]. MSCs in a cell sheet form can secrete extra VEGF and more HGF [41], which is a stronger stimulative clue for angiogenesis. Immunomodulation of MSCs was achieved by secreting cytokines, growth factors, anti-inflammatory mediators, and exosomes [74]. Recent research has shown that MSCs derived from different sources have a similar immunomodulatory mechanism; IFN-γ can activate MSCs and upregulate the secretion of immunomodulation-related molecules such as IDO1, CCL5, CXCL9, CXCL10, CXCL11, CD274, TNSF10, CCL2, and FLT3LG [77]. Another important mechanism is called *cell homing*. It is characterized by a molecular axis resulting from the interaction of the chemokine SDF-1 or CXCl-12 with its specific receptor, the CXCR chemokine receptor type 4 (CXCR-4) [75]. This pathway is influenced by various cytokine and growth factors activated in response to heart tissue regeneration.

Although MSCs have been widely used as therapeutics in cellular therapy, their clinical effectiveness remains limited. MSCs have limited ability to proliferate during in vitro culture. Cellular senescence was observed in MSCs from various sources, such as the bone marrow [78] and dental pulp [79]. MSCs also have a limited lifespan after transplantation [80]. Selection of the cell source is a key point in the application of MSC sheets. MSCs derived from different sources showed similar surface markers and differentiation, but differences remained, such as proliferation and differentiation ability. Efficacy should be evaluated alongside availability and cost. The bone marrow is considered to be the chief source of MSCs, but the availability begins to decrease significantly with the age of the donor. Aging affects the cell subpopulation dynamics and differentiation potential, and the limited potential of the MSCs of aged patients can limit the efficacy of the cellular therapeutic approach [80]. BM-MSCs had decreasing proliferation ability and differentiation potential during an ex vivo expansion [81]. Acquisition of the bone marrow is invasive, and this has led to the discovery of numerous alternative sources. The adipose is a source which could be accessed through liposuction; it is more abundant and accessible than the bone marrow. The isolation of AD-MSCs is the simplest among BM-MSCs, AD-MSCs, and UC-MSCs. However, AD-MSCs may differentiate into chondrocytes after a fifth passage (unpublished data). BM-MSCs and AD-MSCs obtained from aged individuals possess reduced immunomodulatory properties, compared to those from younger donors [82]. UC-MSCs have attracted wide attention among researchers because they represent a bridge between embryonic and adult stem cells and share the same features of all MSCs, such as paracrine functions, immunomodulatory properties, and multi-directional differentiation potential [83]. Additionally, they have the advantage of being able to be isolated and expanded in in vitro culture, offering the possibility of a product. UC-MSCs can be easily obtained *postpartum* and with lesser complications of ethical clearance, as well as self-renewal capacity, than MSCs from other origins. Compared to the complicated separation operation, these advantages are overwhelming. Thus, UC-MSCs are the emerging cell source in cell sheet technology for cardiac tissue regeneration.

SM sheets and iPSC-derived cardiomyocyte sheets have already been used clinically. Autologous SM sheet transplantation can improve cardiac function and avoid rejection [53–55]. However, SM expansion and cell sheet preparation often takes a long time, and it will be limited to patients in urgent need of a transplant. Moreover, SM cell viability may be influenced by individual differences, which makes autologous SM sheets more like a medical technology than a cell therapy product. iPSC-derived cardiomyocytes are the most suitable cell source for cardiac regeneration. Although preclinical studies appear promising, the safety of these artificially generated cells must be evaluated thoroughly before they can be used clinically [49].

## Conclusion and perspective

Many factors influence and modulate the effectiveness of cellular therapy in tissue recovery after damage to ischemic heart tissue, especially the source of the stem cells and the method of stem cell transplantation. Fully automatic processing is a reliable and effective way to eliminate deviation caused by individual operators. In this way, UC-MSC sheets have the potential to become an “off-the-shelf” product with batch-to-batch consistency. The efficiency of UC-MSC sheets is improved because of UC-MSCs as a prospective source. The application of UC-MSC sheets can also convert the use pattern from individual medical technology to a “live medicine,” which could be regulated in a good manufacturing practice (GMP) system. The feasibility and safety of UC-MSC sheets have been tested in animal models of myocardial infarction [33]. More clinical study and clinical trials are necessary to confirm UC-MSC sheets to be a safe and effective therapeutic approach for clinical application.

## Data Availability

Not applicable.

## Abbreviations

3-D: Three-dimensional
AD-MSC: Adipose-derived MSC
BM-MSC: Bone marrow-derived MSC
CMs: Cardiac myocytes
DCM: Dilated cardiomyopathy
ECM: Extracellular matrix
ESC: Embryonic stem cell
GMP: Good manufacturing practice
HGF: Hepatocyte growth factor
HUVEC: Human umbilical vein endothelial cell
ICM: Ischemic cardiomyopathy
iPS-CM: Induced pluripotent stem cell-derived cardiomyocytes
iPSC: Induced pluripotent stem cell
LV: Left ventricular;
LVEF: Left ventricular ejection fraction;
MI: Myocardial infarction
MSC: Mesenchymal stem cell
PIPAAm: Poly(N-isopropylacrylamide)
SM: Skeletal myoblast
UC-MSC: Umbilical cord MSC
VEGF: Vascular endothelial growth factor
